# Cubitus Valgus with Tardy Ulnar Nerve Palsy – Functional Outcome of Milch Osteotomy without Anterior Transposition of Ulnar Nerve

**DOI:** 10.5704/MOJ.2007.021

**Published:** 2020-07

**Authors:** RK Gupta, R Khiyani, KP Majumdar, R Potalia

**Affiliations:** Department of Orthopaedics, Pandit Bhagwat Dayal Sharma Post Graduate Institute of Medical Sciences, Rohtak, India

**Keywords:** non-union, lateral condyle humerus, cubitus valgus, deformity, Milch osteotomy

## Abstract

**Introduction:**

To assess the results of Milch osteotomy in terms of deformity correction and functional outcome in the absence of ulnar nerve transposition.

**Material and Methods:**

Nine patients with cubitus valgus deformity greater than 20° with tardy ulnar nerve palsy (TUNP) operated between 2012 and 2017 were evaluated. Correction by Milch osteotomy and fixation was done in each case, without osteosynthesis of the non-union lateral condyle humerus or transposition of the ulnar nerve. At one year post-operatively, carrying angle, elbow function (Mayo Elbow Performance Score) and ulnar nerve symptoms were assessed.

**Results:**

The mean carrying angle pre-operatively was 30.8° on the affected side which improved to a mean of 8.3° postoperatively with an average correction of 22.5°. The mean elbow flexion pre-operatively was 129.4° which improved to 133.3° post-operatively. The mean preoperative MEP score was 76.7 which improved to a mean of 92.2 post-operatively (p < 0.01). TUNP recovered completely in all the patients.

**Conclusion:**

Milch osteotomy is an effective procedure for cubitus valgus deformity correction and its associated tardy ulnar nerve palsy without a decrease in elbow ROM. Correction of even severe valgus deformities without concurrent anterior transposition of the ulnar nerve is likely to improve ulnar nerve symptoms.

## Introduction

Lateral condyle fracture of the humerus is one of the most common fractures in young children of age around 5-10 years. Non-union or malunion of lateral condyle humerus fractures often present with cubitus valgus deformity and tardy ulnar nerve palsy (TUNP) as a late complication. In such cases, the surgeon has osteosynthesis of the non-union and corrective osteotomy with or without ulnar nerve transposition as possible options.

Many osteotomy techniques and stabilisation methods have been described in the literature with each technique having its advantages and disadvantages^1^. Mortazavi *et al* and Helfet *et al* have reported that anterior transposition of the ulnar nerve is a common surgical technique in patients presenting late with ulnar nerve symptoms secondary to cubitus valgus deformity^2, 3^. However, Barrios *et al*, Dellon *et al*, and Mowlavi *et al*, while advocating anterior transposition of the ulnar nerve observed that the recovery of ulnar nerve symptoms also depends on the severity of pre-operative involvement and correction of the deformity which relaxes the nerve^4-6^. Masada *et al*, have observed that ulnar nerve transposition alone without deformity correction failed to show good results in terms of pain relief or instability^7^. It is rational that the primary cause of TUNP in such cases is a progressive deformity which results in secondary stretching of the nerve. Therefore, it is logical to believe that once the deformity is corrected, the stretch of the nerve should disappear leading to the recovery of neurological symptoms, thereby eliminating the need for transposition.

However, there are only a few reports in the literature indicating results of TUNP with cubitus valgus being managed by corrective osteotomy alone without anterior transposition of the ulnar nerve^8^. We, therefore, reviewed our patients of cubitus valgus deformity with TUNP who underwent Milch osteotomy without any nerve transposition.

The outcome was reviewed regarding final alignment with respect to carrying angle, active elbow ROM, clinical and radiological union and ulnar nerve recovery.

## Materials and Methods

We retrospectively reviewed patients who underwent supracondylar corrective Milch osteotomy between 2012 and 2017 for cubitus valgus deformity and came up with nine patients with TUNP along with cubitus valgus where anterior transposition of the ulnar nerve was not done. Inclusion criteria comprised (i) cubitus valgus deformity at the elbow greater than 20° with TUNP (motor or sensory or both), (ii) post-operative follow-up of minimum one year and (iii) no previous surgical intervention for correction of cubitus valgus deformity or TUNP.

The pre-operative complaints and examination of the elbow in terms of cosmetic appearance, elbow pain, stiffness or instability, range of motion (ROM), carrying angle and ulnar nerve symptoms (Dellon grading), and neurological examination were noted from old medical records^5^. The Milch classification was used to determine whether the fracture passed through (type I) or around (type II) the capitellum^9^.

**Fig. 1:**
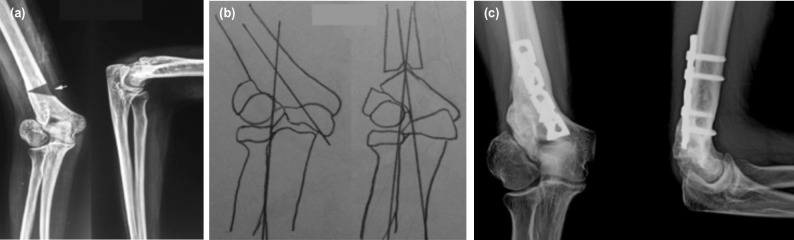
(a) A 18-year-old female patient presented with TUNP secondary to cubitus valgus deformity right side - Pre-operative radiograph showing non-union of the lateral condyle humerus and cubitus valgus deformity (36° valgus). White arrow shows site of planned osteotomy. Black overlaid triangle shows site of planned osteotomy. (b) Milch osteotomy was done to correct the deformity and fixed with plate, without osteosynthesis of the non-union and anterior transposition of ulnar nerve. (c) Six months post-operative radiograph showing union at the osteotomy site and carrying angle corrected to 10° valgus. Excellent results were seen for deformity correction and ulnar nerve symptoms.

Patients were operated in a lateral decubitus position under appropriate anaesthesia using a posterior V-Y triceps flap approach for the distal humerus. The ulnar nerve was exposed, retracted medially and protected throughout the procedure. A simple transverse osteotomy was performed at the intersecting point of the forearm axis with the humeral axis using an oscillating saw (shaded triangle superimposed on the pre-operative radiograph, Fig. 1a). The distal end of the proximal fragment was notched in the middle to receive the apex of the proximal end of the distal fragment. The distal fragment was adducted until the normal carrying angle was restored (Fig. 1b). The fragments were fixed by an appropriate plate and screws in all the cases^10^.

Osteosynthesis of the non-union lateral condyle humerus fracture and anterior transposition of the ulnar nerve was not done in any of the patients. All patients were evaluated on follow-up and the final result was noted at a minimum of one-year post-operatively for carrying angle, elbow function, active ROM, clinical and radiological union, and ulnar nerve recovery. The assessment of ulnar nerve recovery was based on clinical evaluation by grip strength and comparison of sensation with the opposite hand. Mayo Elbow Performance Score (MEP score) was used as an assessment tool for the post-operative outcome and was compared with their pre-op scores^11, 12^.

## Results

There were six males and three females with a mean age of 15.6 years (range 10 to 27 years). The left elbow was affected in seven patients and the right elbow in two patients (all patients had right dominance). Eight of our nine patients had an established non-union of the lateral condyle humerus (four with Milch type I and five with Milch type II), while one patient had malunion.

**Fig. 2:**
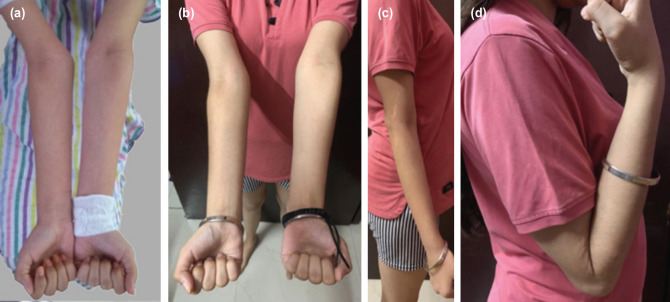
(a) Pre-operative clinical picture of the same patient of Fig. 1. (b) Post-operative frontal view of the same patient showing the deformity correction. (c) Post-operative side view showing full extension. (d) Post-operative side view showing full flexion.

The average duration between fracture and index operation was 38.2 months (range 18 to 90 months). The average postoperative follow-up was 33 months (range 12 to 58 months). The mean pre-operative carrying angle was 30.8° on the affected side (range 23° to 38°) as compared to 9° on the normal side (range 6° to 12°). The mean post-operative carrying angle was 8.3° (range 5° to 10°) with an average correction of 22.5° (Fig. 1c). The improvement in alignment was statistically significant (p < 0.05). The mean preoperative elbow flexion was 129.4° which slightly improved to 133.3° post-operatively (Fig. 2). Average hyperextension of 8.3° seen pre-operatively was almost completely corrected to the neutral position in all patients at 2.8°. Post-operatively the elbow arc of motion was maintained (137.8° pre-op vs 136.1° post-op). The mean pre-operative MEP score was 76.7 which improved to a mean of 92.2 post-operatively (p < 0.01) (i.e. excellent in six patients and good in three patients). All osteotomies healed in a mean time of 46 days (range 34 to 75 days).

The nine patients had various degrees of ulnar nerve involvement at the time of presentation for periods ranging from six months to one year pre-operatively. Six of these patients had paraesthesias and weakness in pinch and grip strength (Dellon’s Grade II) whereas the remaining three patients had paraesthesias, weakness in pinch and grip strength along with intrinsic muscle wasting (Dellon’s Grade III). Post-operatively, all these nine patients had a gradual subjective improvement in terms of paraesthesias, pinch and grip strength which were comparable to their opposite normal hand with a mean time to return to normal after surgery of 4.5 months (range 2 to 8 months). No postoperative complications occurred in any of the patients. Table I shows a summary of the results.

**Table I:** Pre and post-operative highlights of the patients in the study.

				Months since injury		Carrying angle (degrees)		Elbow ROM (degrees)				MEPS		Dellons grade	Recovery of ulnar nerve symptoms
						Pre	Post	Pre		Post		Pre	Post		
								E	F	E	F				
1	19	M	L	60	12	30	8	0	140	0	130	80	95	3	Full
2	12	M	L	24	2	25	5	20	125	0	130	80	95	2	Full
3	14	M	R	30	9	38	7	10	130	0	130	65	80	3	Full
4	12	M	L	24	6	23	7	0	130	5	140	85	00	2	Full
5	10	M	L	20	8	25	10	5	135	0	130	70	85	2	Full
6	12	M	L	18	6	36	10	0	130	0	130	80	95	2	Full
7	18	F	R	48	0	30	10	10	130	5	130	85	00	2	Full
8	16	F	L	30	7	35	0	10	125	0	135	65	85	2	Full
9	27	F	L	90	11	36	8	20	120	10	130	80	95	3	Full

## Discussion

Non-union of lateral condyle humerus fractures can cause progressive cubitus valgus deformity and has been reported to be the most common cause of TUNP^13, 14^.

Reports in literature advocate diverse solutions like corrective osteotomy with or without osteosynthesis of lateral condylar fractures. Most studies, however, advocate the anterior transfer of the ulnar nerve in the presence of TUNP. In the present study, all the patients were managed by Milch osteotomy to correct cubitus valgus but without anterior transfer of ulnar nerve. This was done on account of per-operative observation that after corrective osteotomy the ulnar nerve was found to be lax in its anatomical position itself. Complete recovery of ulnar nerve deficit in all the patients indicates that anterior transposition of ulnar nerve is not required if the basic underlying cause of ulnar nerve deficit i.e. cubitus valgus is rectified by appropriate osteotomy. On account of the number of patients being small and duration of ulnar nerve symptoms not being of long-standing duration in most of the cases, it is not possible to comment on the relation between duration of symptoms and incidence of full neurological recovery. Our results conform with the observations of Bari *et al*, in that correction of deformity does result in the recovery of TUNP without any need for anterior transposition of the ulnar nerve^8^. The observation is in contrast to the studies of Abed *et al* and Kang *et al* where they have advocated anterior transposition of the ulnar nerve in all patients with TUNP^13, 15^.

Similarly, in the study by Mortazavi *et al*, anterior transposition of the ulnar nerve was done in 10 patients for severe TUNP due to cubitus valgus deformity^2^. They reported complete recovery in three patients and symptomatic relief and subjective sensory and motor improvement in the rest with no major complications. However, only four patients among them had cubitus valgus deformity of more than 20°. In the present study, the deformity was more than 20° in all patients (mean = 30.8°) with varying degrees of ulnar nerve deficit, but all the patients recovered completely with the correction of the deformity alone.

Milch osteotomy was preferred over other types of osteotomies as it gives the flexibility of angle of correction even after performing the osteotomy which is not possible in a medial closing wedge or lateral open wedge osteotomies. Osteosynthesis of the lateral condyle of the humerus fractures by K-wires, screws, plate, or tension band wiring has been done by several authors to achieve stability of the lateral column but can result in a significant decrease in the ROM of the elbow apart from other complications like a necrosis of the lateral condyle and persistent non-union^16-18^. Limitations of the present study include its retrospective nature and small sample size.

## Conclusion

Milch osteotomy with stable fixation using plates and screws is a reliable technique for correcting cubitus valgus deformity due to lateral humeral condyle fracture in all patients. Additional anterior transposition of the ulnar nerve may not be required in patients with TUNP as the nerve becomes lax with the deformity correction. However, the recovery of the ulnar nerve may also depend on the duration and severity of the pre-operative involvement. A larger prospective study may still be required to further validate the conclusions given the limited number of cases in the present study.
